# Two new species of doryline ants (Hymenoptera, Formicidae) with 11-segmented antennae from India

**DOI:** 10.3897/zookeys.1056.68722

**Published:** 2021-08-18

**Authors:** Punnath Aswaj, Ramakrishnaiah Sahanashree, Warnakulasuriyage Sudesh Udayakantha, Marathe Aniruddha, Dharma Rajan Priyadarsanan

**Affiliations:** 1 Ashoka Trust for Research in Ecology and the Environment, Royal Enclave, Srirampura, Jakkur Post, Bengaluru – 560064, Karnataka, India Ashoka Trust for Research in Ecology and the Environment Bengaluru India; 2 Department of Zoology and Environmental Management, University of Kelaniya, Sri Lanka University of Kelaniya Kelaniya Sri Lanka; 3 Centre for Ecological Sciences, Indian Institute of Science, Bengaluru – 560012, Karnataka, India Centre for Ecological Sciences, Indian Institute of Science Bengaluru India

**Keywords:** Eastern Himalaya, high elevation, leaf litter, predatory ants, rare ants, West Kameng, Winkler trap

## Abstract

Two new species of the subfamily Dorylinae Leach, 1815 namely *Parasysciaganeshaiahi***sp. nov.** and *Sysciaindica***sp. nov.** are described and illustrated based on the worker caste. These species were collected in the Eaglenest Wildlife Sanctuary, Arunachal Pradesh, Northeast India. Keys to *Parasyscia* of India and *Syscia* of Asia are provided based on the worker caste.

## Introduction

The subfamily Dorylinae Leach, 1815 is a monophyletic group of predatory ants, distributed in the tropical and subtropical regions of the world (Borowiec 2016). It forms the formicoid clade with the subfamilies Aneuretinae, Dolichoderinae, Ectatomminae, Formicinae, Heteroponerinae, Myrmeciinae, Myrmicinae and Pseudomyrmecinae (Ward 2014; Borowiec et al. 2019). The formicoid clade consists of almost 90% of all extant ants (Borowiec et al. 2020). Dorylinae has 733 extant species in 27 genera. The fossil records of the subfamily include one genus and eight species (Bolton 2021). Dorylinae is known to occur on all continents except Antarctica (Borowiec 2016). In India, this subfamily has eight genera and 59 species including four subspecies (Bharti et al. 2016; Bharti et al. 2021; AntWeb 2021).

*Parasyscia* Emery, 1882 and *Syscia* Roger, 1861 are two tropical genera belonging to Dorylinae. *Parasyscia* is a rare ant genus described by Emery with *P.piochardi* Emery, 1882 from Syria as the type species. Later Forel (1892) treated *Parasyscia* as a subgenus of *Cerapachys* and Kempf (1972) synonymised it. Borowiec (2016) revived the status of *Parasyscia* as a valid genus while performing a generic revision of the ant subfamily Dorylinae. The members of this genus are small, cryptic, and nest in decaying logs or under rocks (Brown 1975) while *P.zimmermani* is the only known arboreal species (Sarnat and Economo 2012). This genus consists of 51 valid species, distributed in Afrotropical, Australasia, Indomalaya, Malagasy, Oceania and Palearctic biogeographic regions (AntWeb 2021; Bolton 2021). In India, seven species of the genus *Parasyscia* are recorded: *Parasysciaaitkenii* (Forel, 1900); *P.indica* (Brown, 1975); *P.keralensis* (Karmaly, 2012); *P.browni* (Bharti & Wachkoo, 2013); *P.schoedli* (Bharti & Akbar, 2013); *P.seema* (Bharti & Akbar, 2013) and *P.wighti* (Bharti & Akbar, 2013). *Parasysciaaitkenii*, reported from Meghalaya, is the only species known from Northeast India (Bharti et al. 2016) so far. The species is also reported from Goa, Haryana, Karnataka, Kerala, Punjab and West Bengal. *Parasysciabrowni* is known from Himachal Pradesh and Uttarakhand while *P.indica*, *P.keralensis*, *P.schoedli*, *P.seema* and *P.wighti* are reported from Kerala (Bharti et al. 2016). All species except *P.aitkenii* are endemic to India. The present discovery of *P.ganeshaiahi* sp. nov. marks the first record of the genus *Parasyscia* from the state Arunachal Pradesh, Northeast India. *Syscia* is a rare ant genus erected by Roger (1861) in his paper on Ponera-like ants, with the Sri Lankan species *S.typhla* Roger, 1861 as the type species. Forel (1900) and Bingham (1903) treated *Syscia* as a valid genus while Wheeler (1902) and Emery (1911) considered it as a subgenus of *Cerapachys* Smith, 1857. Later Kempf (1972) treated *Syscia* as a junior synonym of *Cerapachys*. However, after molecular studies on Dorylinae, Borowiec (2016) recognized *Syscia* as a valid genus. Currently, this genus has 38 valid species (Longino and Branstetter 2021). These rare ants are usually encountered in leaf litter, rotting wood, and soil habitats (Jaitrong et al. 2020). Members of the genus *Syscia* are distributed in the New and Old Worlds (Borowiec 2016; AntWeb 2021). In Asia, the genus has been known from China, Japan, Sri Lanka and Thailand (AntWeb 2021). The literature record shows the presence of *Sysciatyphla* in India as *Cerapachystyphlus* (Ghosh et al. 2007). However, this species was not listed in an updated checklist of the Indian ants by Bharti et al. (2016) and global ant database AntWeb (2021). Hence the present study is the first verified record of the genus *Syscia* from India.

We have collected a single worker specimen belonging to each of the genera *Parasyscia* and *Syscia* in the Eaglenest Wild Life Sanctuary, Arunachal Pradesh, Northeast India in 2013. Even though we have performed an extensive collection in the region with Winkler traps, pitfall traps and hand picking, we could not find more than one specimen of the aforementioned species. This suggests that such subterranean ants are rarely encountered in the field and are therefore hard to collect. A considerable number of such rare ants are known based on a single specimen and, provided they are significantly different in morphology from closely related species, warrant new species description. Keys to all known valid species of *Parasyscia* of India and *Syscia* of Asia are also provided based on the worker caste.

## Materials and methods

**Figure 1.**
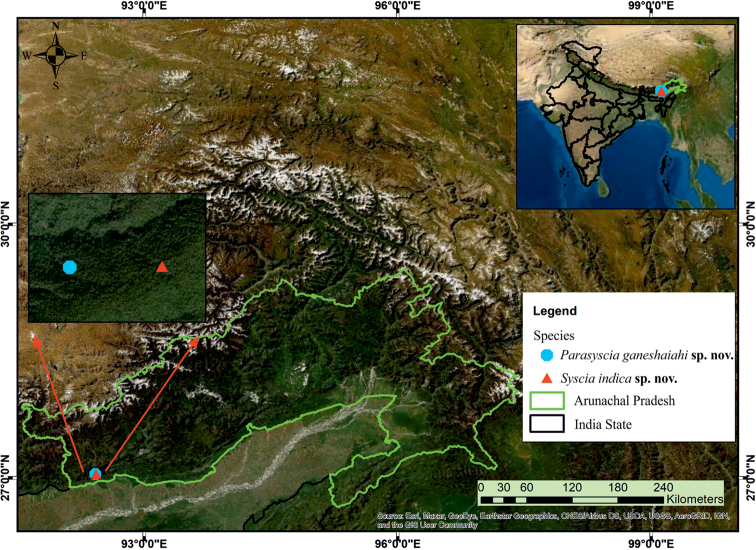
Map showing the type localities of *Parasysciaganeshaiahi* sp. nov. and *Sysciaindica* sp. nov. in the Eaglenest Wildlife Sanctuary, Arunachal Pradesh, Northeast India (Source: Esri, Maxar, GeoEye, Earthstar geographics, CNES/Airbus DS, USDA, USGS, AeroGRID, IGN, and the GIS User Community).

A worker specimen of *Parasyscia* and *Syscia* were collected in the Eaglenest Wildlife Sanctuary, West Kameng District of Arunachal Pradesh, an Indian state in the Himalayan foothills (Fig. 1). The specimens were collected from a high elevation leaf litter sample using Winkler extractors. The collected specimens were preserved in alcohol before mounting. The point mounted specimens were examined under a Zeiss SteREO Discover.V8 microscope. We compared our specimens with the images of closely similar species such as *P.piochardi* Emery, 1882, *S.chaladthanyakiji* Jaitrong, Wiwatwitaya & Yamane, 2020 and *S.typhla* Roger, 1861. The images of *P.piochardi* were accessed on AntWeb.org (2021, CASENT0281973, photographed by Michele Esposito, California Academy of Sciences). Similarly, the type images of *S.typhla* were accessed on AntWeb.org (2021, FOCOL0804, California Academy of Sciences) while the holotype images of *S.chaladthanyakiji* were obtained from Jaitrong et al. (2020). The specimens were imaged at 200× magnification and extended focus montage images were taken using a Keyence VHX 6000 digital microscope. Artefacts and unnecessary parts of the images were removed and aligned into a plate using Adobe Photoshop CC 2019. A distribution map of the newly described species was prepared using ArcGIS 10.4.1 (ArcGIS 2021). Body measurements were taken with AxioVision 4.8 (Carl Zeiss, Germany). The holotype specimens of both species are deposited in the National Bureau of Agricultural Insect Resources (**ICAR-NBAIR**), Bangalore, India. Measurements and morphological terminology follow Jaitrong et al. (2020) and Sharaf et al. (2018).

**BL** Body length: total length of body calculated by summing lengths of head, mesosoma, petiole and gaster;

**HL** Head length: maximum length of head in full-face view, from transverse line spanning the anterior-most points of clypeus to that of posteriormost points of head;

**HW** Head width: maximum width of head in full-face view (excluding eyes);

**EL** Eye length: maximum length of eye;

**TL** Tempora length: measured from posterior margin of eye to posteromedian margin of head capsule;

**SL** Scape length: maximum length of antennal scape excluding condyle;

**PRW** Pronotal width: maximum width of pronotum in dorsal view;

**WL** Weber’s length: diagonal length of mesosoma in profile, from anterior-most point of pronotal slope (excluding the neck) to posteroventral margin of propodeal lobe;

**PL** Petiolar length: maximum length of petiole in lateral view (excluding helcium);

**PH** Petiolar height: maximum height of petiole (including subpetiolar process);

**PW** Petiolar width: maximum width of petiole in dorsal view;

**PPL** Postpetiolar length: maximum length of postpetiole in lateral view (excluding helcium);

**PPH** Postpetiolar height: maximum height of postpetiole in lateral view;

**PPW** Postpetiolar width: maximum width of postpetiole in dorsal view;

**CI** Cephalic index: HW/HL × 100;

**SI** Scape index: SL/HW × 100;

**EI** Eye index: EL/HW × 100;

**PI1** Petiolar index 1: PL/PH × 100;

**PI2** Petiolar index 2: PW/PL × 100;

**PPI1** Postpetiolar index 1: PPL/PPH × 100;

**PPI2** Postpetiolar index 2: PPW/PPL × 100;

**WI** Waist index: PPW/PW × 100.

## Results

### Parasyscia

Taxon classificationAnimaliaHymenopteraFormicidae

Emery, 1882

1317CA20-AA4B-5C1E-BD96-5F80E11BEBDE

Parasyscia Emery, in André, 1882c: 235. Type species: Parasysciapiochardi, by monotypy.
Parasyscia subgenus of Cerapachys : Forel 1892l: 243.
Parasyscia junior synonym of Cerapachys: Kempf 1972a: 76.
Parasyscia as genus: Borowiec 2016: 198.

#### Diagnosis.

*Parasyscia* workers can be identified by the following combination of characters: 1) propodeal spiracle positioned low on the sclerite and propodeal lobes present; 2) presence of a constriction between abdominal segments III and IV; 3) petiole dorsolaterally not marginate; 4) constriction between abdominal segments IV, V, and VI not present; 5) pronotomesopleural suture fused; 6) helcium axial; 7) middle tibiae with a single pectinate spur; 8) pretarsal claws unarmed; 9) abdominal segment III anterodorsally often marginate (Borowiec 2016).

### Parasyscia ganeshaiahi sp. nov.

Taxon classificationAnimaliaHymenopteraFormicidae

0496DD0B-4255-5257-B70F-3536B2A13A1E

http://zoobank.org/4E992EB2-10F9-4CCD-A1EB-45E5F9D55915

Fig. 2A–F

#### Material examined.

***Holotype*** One worker, point mounted. Original label: “India, Arunachal Pradesh, West Kameng, Eaglenest WLS, 27.0433°N, 92.4209°E, 1400m, Winkler extraction method, 14^th^ April 2013, Aniruddha Marathe leg.” [NBAIR/HYM-FOR/1721-1].

#### Worker description.

**Figure 2.**
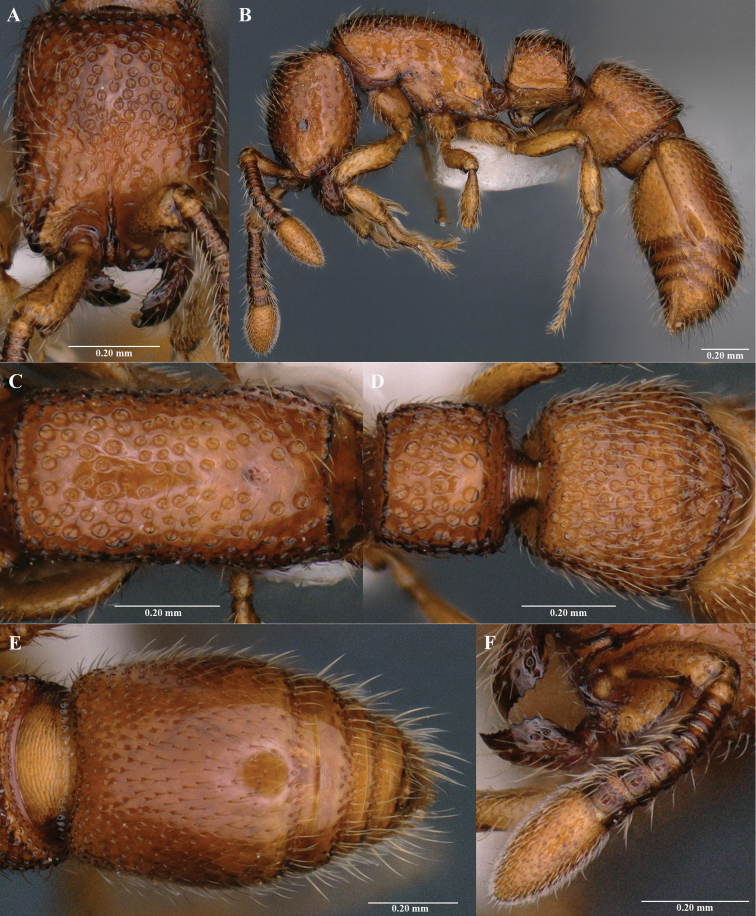
*Parasysciaganeshaiahi* sp. nov., holotype worker **A** head in full-face view **B** body in profile view **C** mesosoma in dorsal view **D** petiole and postpetiole in dorsal view **E** girdling constriction and gaster in dorsal view **F** antenna and Mandibles.

Measurements and indices (holotype): BL 2.82; HL 0.58; HW 0.46; EL 0.05; TL 0.31; SL 0.22; PRW 0.32; WL 0.79; PL 0.21; PH 0.39; PW 0.30; PPL 0.39; PPH 0.44; PPW 0.45. Indices. CI 79; EI 11; SI 48; PI1 54; PI2 143; PPI1 89; PPI2 115; WI 150 (all measurements in mm). ***Head*.** In full-face view, rectangular, distinctly longer than broad; lateral margin weakly convex; posterior margin weakly concave (Fig. 2A). Antennae 11 segmented; scape short, when folded back fail to reach posterior margin of eyes; Antennal segment II almost as long as broad; segments III–X distinctly broader than long; apical segment (XI) swollen forming a distinct club, 2×longer than IX–X combined (Fig. 2F). Frontal carinae short, united and slightly extended behind the antennal socket. Eyes small; located slightly anterior to the mid-length of head (Fig. 2A). Mandibles triangular; masticatory margin with a row of denticles (Fig. 2F). ***Mesosoma*.** In dorsal view, elongate with almost parallel lateral sides (Fig. 2C). In profile view, dorsal outline weakly convex; promesonotal suture and metanotal groove absent (Fig. 2B). Propodeal declivity in profile view, lightly concave; declivous face with distinct carina across the top and along the lateral margins; propodeal lobe in profile view, strongly convex (Fig. 2B, C). ***Metasoma*.** Petiole in dorsal view, distinctly broader than long; anterior and lateral margin with distinct carina; anterior and posterior margins transverse (Fig. 2B, D). In profile view, petiole shorter than high with weakly convex dorsal outline. Postpetiole in dorsal view, larger than petiole; broader than long; strongly convex posterior margin (Fig. 2D). In profile view, postpetiole shorter than high; dorsal outline weakly convex (Fig. 2B). Abdominal tergite IV (first gastral tergite) in dorsal view, elongate, occupying most part of gaster; anterior margin strongly concave; lateral sides strongly convex. Base of cinctus of first gastral tergite with cross ribs (Fig. 2E). ***Sculpture*.** Body with numerous distinct foveolae with smooth interspaces. Propodeal dorsal surface medially with a distinct unsculptured area. Postpetiole with more closely-spaced foveolae. First gastral tergite anteriorly with large foveolae and posteriorly with smaller foveolae. Remaining gastral segments anteriorly smooth and posteriorly with small foveolae.

***Pilosity*.** Body covered with erect to sub-erect hairs. Sides of head and legs with relatively shorter hairs. Apical antennal segment with abundant short decumbent hairs. *Body coloration*. Body mainly yellowish brown. Antennal segment III to X and mandibles reddish brown.

#### Recognition.

*Parasysciaganeshaiahi* sp. nov. is similar to the Palearctic *P.piochardi* Emery, 1882 in general appearance and 11-segmented antennae. *Parasysciapiochardi* is known from Israel, Lebanon, Syrian Arab Republic and Turkey (AntWeb 2021; Bolton 2021). *Parasysciaganeshaiahi* is distinguished from *P.piochardi* by the following characteristics: 1) head in full-face view, rectangular with weakly convex lateral sides in *P.ganeshaiahi* (head nearly oval shaped with strongly convex lateral sides in *P.piochardi*); 2) strong sculpturing with relatively larger foveolae in *P.ganeshaiahi* (weakly sculptured with shallow, small foveolae in *P.piochardi*); 3) head in full-face view, fovea larger in size and comparatively closely spaced in *P.ganeshaiahi* (fovea smaller with distinct unsculptured area present in *P.piochardi*); 4) frontal carinae united posteriorly and extended behind in *P.ganeshaiahi* (frontal carinae not united and extended behind in *P.piochardi*); 5) mesosoma in dorsal view, with nearly straight lateral margins in *P.ganeshaiahi* (mesosoma in dorsal view, medially concave in *P.piochardi*); 6) petiole in profile view, with straight anterior slope in *P.ganeshaiahi* (weakly convex anterior margin in *P.piochardi*); 7) anterior margin of petiole in dorsal view, with a distinct carina in *P.ganeshaiahi* (carina absent in *P.piochardi*); 8) *P.ganeshaiahi* is a smaller species, HW 0.46, TL 2.82 in (*P.piochardi* is larger, HW 0.53, TL 3.40); 9) *P.ganeshaiahi* has comparatively bigger eyes in relation to head width, EI 11 (*P.piochardi* has smaller eyes in relation to the head width, EI 6).

#### Habitat.

The type specimen was collected from the Eaglenest Wildlife Sanctuary at an elevation of 1400 m above sea level. The collection site has a canopy cover of about 90% with low light penetration. The soil had a pH of 6.83 and temperature 17°C at the time of collection. A single worker specimen was collected using a Winkler extractor from sifted leaf litter taken from 1 m^2^, which accounted for 760 ml of leaf litter. We captured three additional ant genera (*Aphaenogaster*, *Carebara* and *Paratrechina*) in the same trap.

#### Distribution.

Indomalaya: India (Arunachal Pradesh).

#### Etymology.

With 2021 being the 25^th^ anniversary of ATREE, this species is a Latin noun in the genitive case named in honour of one of its founders, Prof. K. N. Ganeshaiah, eminent ecologist and writer, who was instrumental in establishing Insect Taxonomy and Conservation Laboratory in ATREE.

### Key to *Parasyscia* of India based on the worker caste

Modified after Bharti and Akbar 2013.

**Table:** 

1	Antenna 11-segmented (Fig. 1F)	***P.ganeshaiahi* sp. nov.**
–	Antenna 12-segmented	**2**
2	Cephalic dorsum with rugo-reticulate sculpture	***P.browni* (Bharti & Wachkoo, 2013)**
–	Cephalic dorsum with either small punctures or foveae	**3**
3	Punctures on dorsum of head relatively small, their diameter smaller than the average distance separating them	**4**
–	Punctures on head dorsum large, foveiform, dense, their diameter as large, or larger than, the average distance separating them, and in most cases these are contiguous	**5**
4	Shiny species; body sculpture reduced; eyes breaking the lateral margins of head; colour varies from light orange to dark red	***P.schoedli* (Bharti & Akbar, 2013)**
–	Dull coloured species; body sculpture prominent; eyes not breaking the lateral margins of head; colour brown to dark brown	***P.seema* (Bharti & Akbar, 2013)**
5	Eyes reduced (EL < 0.1 mm)	***P.wighti* (Bharti & Akbar, 2013)**
–	Eyes large (EL > 0.2 mm)	**6**
6	Head reddish brown or red; trunk and both nodes red; gaster black or dark brown; dorsal surface of mesosoma densely and finely sculptured; foveate or rugo reticulate	***P.aitkenii* (Forel, 1900)**
–	Body unicolorous, lighter brownish red; dorsal surface of mesosoma mostly smooth with few scattered punctures along sides	***P.indica* (Brown, 1975)**

**Note**: *Parasysciakeralensis* (Karmaly, 2012) is highly dubious and considered as a species inquirenda (Bharti and Akbar 2013). Hence *P.keralensis* is not included in this key.

### Syscia

Taxon classificationAnimaliaHymenopteraFormicidae

Roger, 1861

81715D1C-67EC-54D2-A7B6-010B60F5D2D1

Syscia Roger, 1861a 19. Type species: Sysciatyphla, by monotypy.
Syscia subgenus of Cerapachys : Wheeler, W.M. 1902d: 185; Emery 1902c: 24.
Syscia senior synonym of Cysias: Emery 1911d: 10.
Syscia junior synonym of Cerapachys: Kempf 1972a: 76.
Syscia as genus: Borowiec 2016: 219.

#### Diagnosis.

Borowiec (2016) defined this genus as follows: 1) 11- or 9-segmented antennae; 2) eyes small to absent; 3) body usually heavily sculptured with abundant pilosity; 4) uniformly coloured body, ranges from yellow through reddish to dark brown but never black; 5) basal segment of hind tarsus widening distally with a light patch of cuticle on the inner (flexor) side; 6) abdominal tergite IV anteriorly folding over sternite.

### Syscia indica sp. nov.

Taxon classificationAnimaliaHymenopteraFormicidae

6803E4A5-91CF-5A69-8FD4-306B53CF8AA9

http://zoobank.org/3A9C67DF-8692-40EF-A3FD-3102D10C1253

Fig. 3A–F

#### Material examined.

***Holotype*** One worker, Point mounted. Original label: “India, Arunachal Pradesh, West Kameng, Eaglenest WLS, 27.0434°N, 92.4302°E, 1600 m, Winkler extraction method, 10.IV.2013, Aniruddha Marathe leg.” [NBAIR/HYM-FOR/1721-2].

#### Worker description.

Measurements and indices (holotype): BL 3.33; HL 0.68; HW 0.50; SL 0.25; PRW 0.35; WL 0.85; PL 0.36, PH 0.31, PW 0.29, PPL 0.41, PPH 0.47, PPW 0.43. Indices: CI 74, SI 50, PI1 116, PI2 81, PPI1 87, PPI2 105, WI 148 (all measurements in mm).

**Figure 3.**
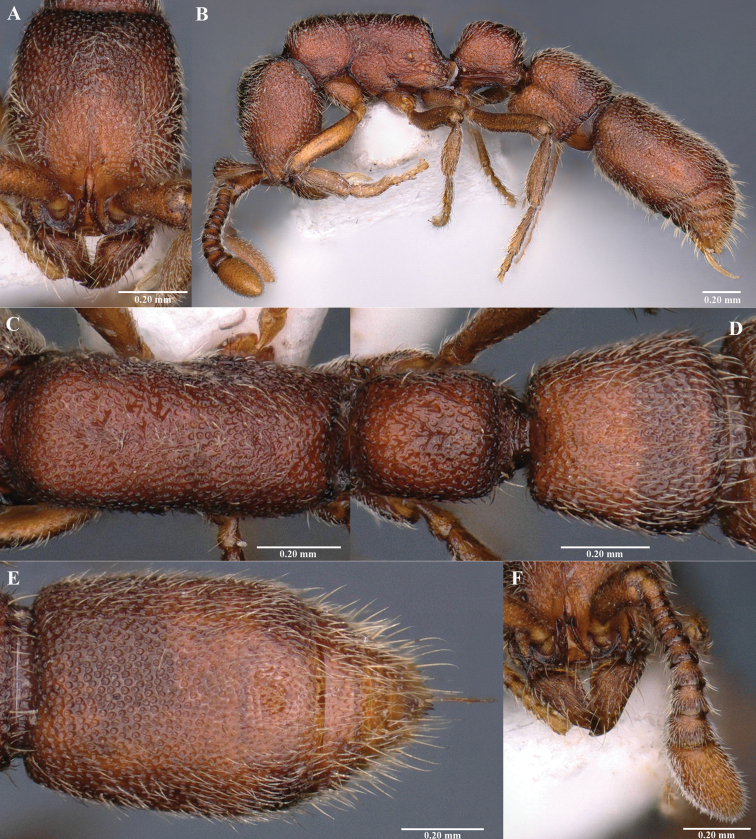
*Sysciaindica* sp. nov., holotype worker **A** head in full-face view **B** body in profile view **C** mesosoma in dorsal view **D** petiole and postpetiole in dorsal view **E** girdling constriction and gaster in dorsal view **F** antenna and Mandibles.

***Head*.** In full-face view, rectangular, distinctly longer than broad; lateral margin weakly convex; posterior margin weakly concave (Fig. 3A). Antennae 11-segmented, apical segment (XI) longer than IX–X combined; scape short, reaching mid-length of head when folded back. Antennal segment II almost as long as broad, segments III–X slightly broader than long (Fig. 3F). Frontal carinae short and narrow, reaching less than half of head length. Eyes and ocelli completely absent (Fig. 3A). Mandibles triangular; masticatory margin serrated, without distinct teeth (Fig. 3F). ***Mesosoma*.** In dorsal view, elongate with almost parallel lateral sides (Fig. 3C). In profile view, dorsal outline weakly convex; promesonotal suture and metanotal groove absent; mesopleuron demarcated from propleuron by a distinct pronotomesopleural suture; mesopleuron not clearly differentiated from metapleuron. Propodeal declivity feebly concave, encircled with a distinct thin rim (Fig. 3B). ***Metasoma*.** In dorsal view, petiole distinctly longer than broad; almost parallel sided (Fig. 3D). In profile view, petiole subrectangular; slightly longer than high with weakly convex dorsal outline. Subpetiolar process subrectangular; ventral outline strongly concave (Fig. 3B). Postpetiole in dorsal view, larger than petiole; slightly longer than broad (Fig. 3D). In profile view, postpetiole slightly shorter than high; dorsal outline weakly convex. Postpetiolar sternite in profile view low, ventral margin weakly convex; anteroventrally produced into a blunt angle (Fig. 3B). Abdominal tergite IV (first gastral tergite) in dorsal view, elongate with anterior margin weakly concave; lateral sides weakly convex (Fig. 3E). Base of cinctus of first gastral tergite with cross ribs (Fig. 3E). ***Sculpture*.** Body with numerous, relatively small, closely-spaced foveolae. Ventrolateral surface of petiole with two distinct almost parallel carinae just above the subpetiolar process. Antennal scape, outer surface of mandible and legs with fine dense micropunctures. ***Pilosity*.** Body covered with erect and sub-erect hairs. Dorsum of postpetiole and first gastral tergite with sparse erect hairs mixed with dense long decumbent hairs. Antennae and legs with dense short decumbent hairs. *Body coloration*. Body reddish brown. Antennae and legs yellowish brown.

#### Recognition.

*Sysciaindica* sp. nov. is similar to *S.chaladthanyakiji* in general appearance and 11-segmented antennae. However, *S.indica* can be separated from *S.chaladthanyakiji* by the following characteristics: 1) weak sculpture with relatively small and closely-spaced foveolae in *S.indica* (stronger sculpturing with well-defined macropunctures in *S.chaladthanyakiji*); 2) abdominal segment IV (first gastral tergite) with large number of closely-spaced foveolae in *S.indica* (first gastral tergite with lesser number of relatively widely spaced foveolae in *S.chaladthanyakiji*); 3) remaining gastral segments with feeble foveolae in *S.indica* (remaining gastral segments anteriorly with an unscupltured area in *S.chaladthanyakiji)*; 4) head in full-face view oval shaped with convex lateral sides and moderately concave posterior margin in *S.indica* (head in full-face view rectangular, almost parallel sides, posterior margin weakly concave in *S.chaladthanyakiji*); 5) CI 74, SI 50 in *S.indica* (CI 80–84, SI 56–63 in *S.chaladthanyakiji)*. *Sysciaindica* sp. nov. is also similar to the Sri Lankan and Chinese *S.typhla* in general appearance. However, the new species can be easily separated from *S.typhla* by the 1) presence of 11-segmented antennae (9-segmented in *S.typhla*); 2) foveolae on cephalic dorsum smaller in diameter and closely spaced in *S.indica* (foveolae comparatively larger in diameter and widely spaced in *S.typhla*); 3) posterior cephalic margin nearly transverse in *S.Indica* (posterior cephalic margin strongly concave in *S.typhla*); 4) base of cinctus of first gastral tergite with cross ribs in *S.indica* (base of cinctus of first gastral tergite without cross ribs in *S.typhla*).

#### Habitat.

The holotype was collected from the Eaglenest Wildlife Sanctuary at an elevation of 1600 m above sea level. The region has a canopy cover of about 85% and no indications of anthropogenic disturbances. The soil had a pH of 6.85 and temperature 16°C at the time of collection. The specimen was collected using a Winkler extractor from sifted leaf litter of 1 m^2^. We were able to capture one additional ant genus *Carebara* in the same trap, which consisted of 295 ml leaf litter.

#### Distribution.

Indomalaya: India (Arunachal Pradesh).

#### Etymology.

The specific epithet indica is a Latin singular feminine adjective in the nominative case and refers to the country where the species was collected.

### Key to *Syscia* of Asia

**Table:** 

1	Antennae 9-segmented	***S.typhla* Roger, 1861**
–	Antennae 11-segmented	**2**
2	Body surface opaque with fine reticulations	***S.humicola* (Ogata, 1983)**
–	Body surface with well-defined punctures or foveolae	**3**
3	Body entirely covered with deep foveae; WI 110–122	***S.reticularis* Jaitrong, Wiwatwitaya & Yamane, 2020**
–	Body with numerous punctures or foveolae; WI ≥ 130	**4**
4	Weak sculpture with relatively small and closely-spaced foveolae (Fig. 2); CI 74, SI 50	***S.indica* sp. nov.**
–	Stronger sculpturing with well-defined macropunctures; CI 80–84, SI 56–63	***S.chaladthanyakiji* Jaitrong, Wiwatwitaya & Yamane, 2020**

## Discussion

*Parasyscia* and *Syscia* are rare ants belonging to the subfamily Dorylinae which were previously included in the genus *Cerapachys*. Recent phylogenetic studies have revived the status and considered them as valid genera. Here we describe two new species of doryline ants one in each of these genera, based on the worker caste. A single worker specimen of *P.ganeshaiahi* sp. nov. and *S.indica* sp. nov. were collected using Winkler extractors in the Eaglenest Wildlife Sanctuary in Northeast India. The Eaglenest Wildlife Sanctuary has an area of 218 km^2^ and the elevation ranges from 500 m to 3250 m. It has a wide range of forests starting from tropical evergreen forests in the lower elevation, to temperate broad-leaved forest in the mid-elevation to rhododendrons and conifers in the higher altitudes. The protected area lies in a region of Himalayas with exceptional biodiversity. The present discovery of two new species of doryline ants shows the importance of exploring the Eastern Himalayas especially by using non-conventional extraction methods such as Winkler traps. The Winkler extraction method is exceptionally good for collecting rarely sampled subterranean ants from the leaf litter. The discovery of *P.ganeshaiahi* sp. nov. and *S.indica* sp. nov. is an important contribution to the understanding of the Indian myrmecofauna. *Parasysciaganeshaiahi* sp. nov. marks the first record of a *Parasyscia* species with 11-segmented antennae from India. All other seven known species of the Indian *Parasyscia* have 12-segmented antennae (Bharti and Akbar 2013). It also marks the first record of the genus *Parasyscia* from Arunachal Pradesh. Although literature records show the presence of *S.typhla* in India as *Cerapachystyphlus* (Ghosh et al. 2007), the species lacks a verified museum record. Considering data in the global ant database (AntWeb 2021) and checklist of the Indian ants (Bharti et al. 2016), the previous record of *S.typhla* in India is highly dubious and needs to be verified with specimen records. Hence, the present discovery is the first confirmed record of the genus from India. In the Indomalaya region, *Syscia* has only three known species. Though we have performed an extensive sampling across northeastern states in India using pitfall and Winkler traps, we were not able to find additional specimens of *P.ganeshaiahi* and *S.indica*. Based on the rarity and significant character differences from other known species, we can undoubtedly consider them as valid species.

## Supplementary Material

XML Treatment for Parasyscia

XML Treatment for Parasyscia ganeshaiahi

XML Treatment for Syscia

XML Treatment for Syscia indica
